# Economic evaluation of access to musculoskeletal care: the case of waiting for total knee arthroplasty

**DOI:** 10.1186/1471-2474-15-22

**Published:** 2014-01-18

**Authors:** Richard C Mather, Kevin T Hug, Lori A Orlando, Tyler Steven Watters, Lane Koenig, Ryan M Nunley, Michael P Bolognesi

**Affiliations:** 1 Department of Orthopaedic Surgery, Duke University Medical Center, Durham, NC 27710, England; 2 Department of Orthopaedics and Sports Medicine, University of Washington, Seattle, WA 98195, USA; 3 Department of Medicine, Duke University Medical Center, Durham, NC 27710, England; 4 KNG Health Consulting, Rockville, MD 20850, USA; 5 Department of Orthopedic Surgery, Washington University School of Medicine, St. Louis, MO 63110, USA

**Keywords:** Total knee arthroplasty, Cost-effectiveness analysis, Cost-utility analysis, Markov, Decision analysis, Healthcare economics

## Abstract

**Background:**

The projected demand for total knee arthroplasty is staggering. At its root, the solution involves increasing supply or decreasing demand. Other developed nations have used rationing and wait times to distribute this service. However, economic impact and cost-effectiveness of waiting for TKA is unknown.

**Methods:**

A Markov decision model was constructed for a cost-utility analysis of three treatment strategies for end-stage knee osteoarthritis: 1) TKA without delay, 2) a waiting period with no non-operative treatment and 3) a non-operative treatment bridge during that waiting period in a cohort of 60 year-old patients. Outcome probabilities and effectiveness were derived from the literature. Costs were estimated from the societal perspective with national average Medicare reimbursement. Effectiveness was expressed in quality-adjusted life years (QALYs) gained. Principal outcome measures were average incremental costs, effectiveness, and quality-adjusted life years; and net health benefits.

**Results:**

In the base case, a 2-year wait-time both with and without a non-operative treatment bridge resulted in a lower number of average QALYs gained (11.57 (no bridge) and 11.95 (bridge) vs. 12.14 (no delay). The average cost was $1,660 higher for TKA without delay than wait-time with no bridge, but $1,810 less than wait-time with non-operative bridge. The incremental cost-effectiveness ratio comparing wait-time with no bridge to TKA without delay was $2,901/QALY. When comparing TKA without delay to waiting with non-operative bridge, TKA without delay produced greater utility at a lower cost to society.

**Conclusions:**

TKA without delay is the preferred cost-effective treatment strategy when compared to a waiting for TKA without non-operative bridge. TKA without delay is cost saving when a non-operative bridge is used during the waiting period. As it is unlikely that patients waiting for TKA would not receive non-operative treatment, TKA without delay may be an overall cost-saving health care delivery strategy. Policies aimed at increasing the supply of TKA should be considered as savings exist that could indirectly fund those strategies.

## Background

Total knee arthroplasty (TKA) is an effective treatment to alleviate pain and improve physical functioning in patients with arthritic knees [1]. Approximately 300,000 TKAs are performed in the United States each year making it an exceptionally common surgical procedure [2]. As the population continues to age, the demand for primary TKAs is projected to increase 673% by 2030 [3]. Others have projected significant workforce shortages poised to meet this growing demand [4,5]. Undoubtedly, great challenges about how to narrow this gap in supply and demand are upon us.

Strategies to meet this demand include workforce increases, operational efficiency gains and minimizing revisions. However, experience from other developed countries as well as the U.S. Veterans Administration Hospital system suggest wait times will increase rapidly as demand rises. The current mean wait time for TKA in Canada is 237 days and the mean wait time for TKA within the Veterans Affairs Healthcare System in the United States can be as long as two years [6,7]. In particular the wait time in Canada has grown by 423% since the mid-1990s. Furthermore, increased waiting time for TKA has the potential to negatively impact patient outcome. Wait times longer than 6 months may reduce health-related quality of life and increase contralateral knee pain 6 months after TKA [8,9].

TKA is known to be a cost-effective treatment across populations in the United States and abroad [10-12]. Furthermore, end-stage arthritis is associated with high direct medical costs and healthcare resource utilization [13,14]. However, the societal economic impact of wait times for elective procedures remains unclear. A complete understanding of the entire care pathway of end-stage OA is necessary to direct policymaking and optimizes resource allocation. Using the power of decision analysis, the purpose of this study was to explore the societal economic impact of wait times for primary TKA.

## Methods

### General model overview

The decision model and analysis in this study was performed in accordance with the consensus-based recommendations for the conduct of cost-effectiveness analysis advocated by the Panel on Cost-Effectiveness in Health and Medicine [15-17]. The model compared three possible treatment arms for a patient age 60 with end-stage knee OA requiring TKA. Two scenarios were examined in the base case, (A) direct medical costs only and (B) direct + indirect costs. The analysis was performed with a decision tree using a general decision analysis software package (TreeAge Pro Suite 2011; TreeAge Software Inc., Williamstown, Massachusetts).

### Decision model

**Figure 1:**
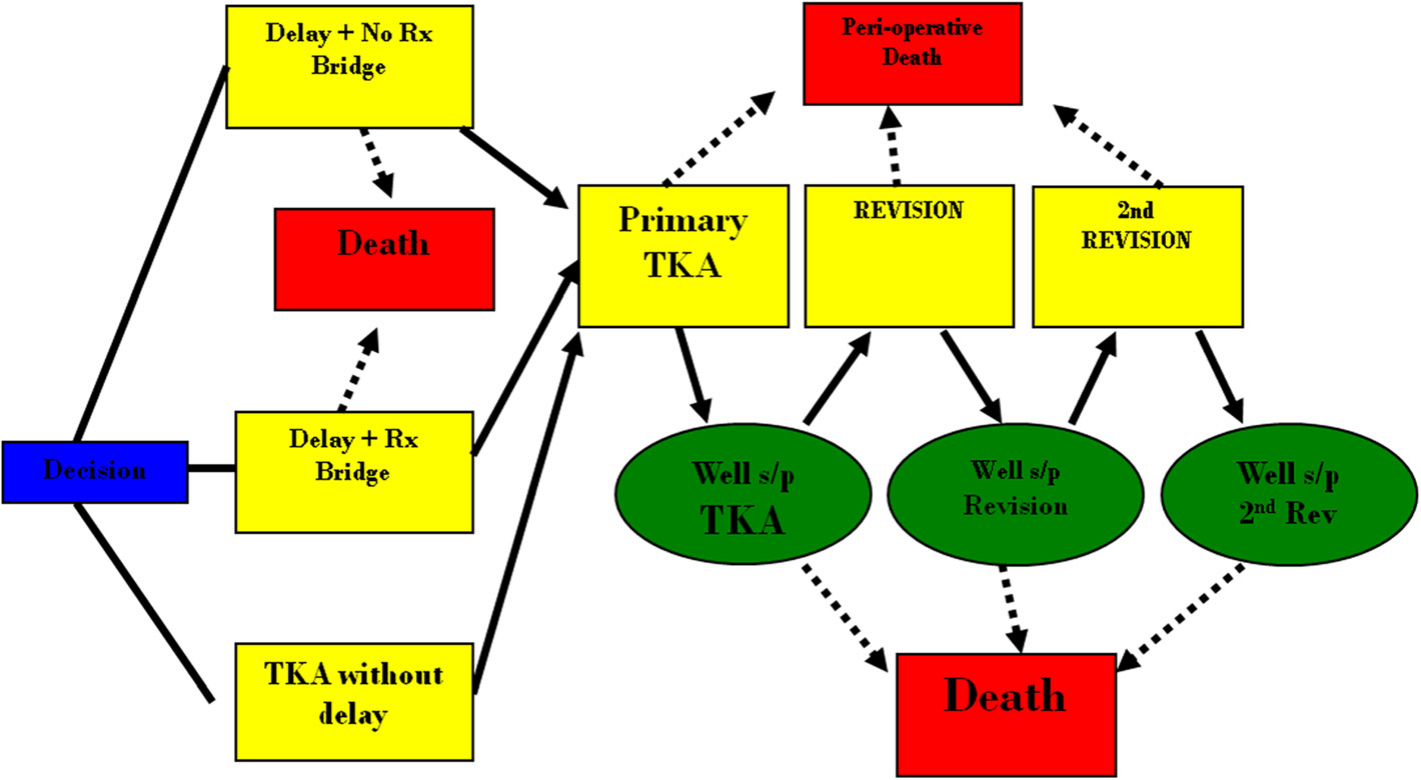
**Health state diagram.** The figure shows the health state diagram used to model patients undergoing either TKA without delay, delayed TKA with non-operative bridge treatment, or delayed TKA with no bridge treatment. The diagram reflects the distinct health states in the model.

A Markov health state decision model was created for the treatment of end-stage knee OA [18]. The decision tree consists of three principal treatment arms, or strategies: TKA without delay, delayed TKA with non-operative bridge treatment and delayed TKA with no bridge treatment. A simplified schematic of the model is shown in Figure 1. Both treatment arms and transition probabilities are consistent in design to those previously published in the literature [19-22].

After undergoing TKA individuals remain in an initial post-procedure state for one month, either surviving or dying based on all cause or perioperative mortality (Table 1). Perioperative complications were not directly modeled. No revisions can occur until the end of year one. All patients then transition to well TKA, representative of the average outcome after TKA. This cohort of patients will subsequently remain in TKA until they die of natural causes or undergo revision TKA. Patients can undergo two revision TKAs.

**Table 1:** Model parameters

**Health state utility (QALY)**	**Base case**	**Sensitivity analysis**
End-stage Knee OA	0.6	0.4-0.8
End-stage knee OA with treatment bridge	0.7	0.6-0.9
Primary TKA	0.9	0.6-0.9
Revision TKA	0.85	0.6-0.9
Recovery from revision TKA	-0.1	-0.25-0
Recovery from early TKA complication	-0.20	-0.40-0
Transition probabilities		
Early complication after TKA	0.01	0-0.05
Failure to revision of primary TKA (annual)		
Years 0-9	0.005	0-0.02
Years 10-19	0.01	0-0.05
Years 20+	0.02	0-0.05
Failure to revision of revision TKA (annual)		
Years 0-9	0.01	0-0.04
Years 10-19	0.02	0-0.08
Years 20+	0.04	0-0.10
All-cause mortality	Life Tables	None
30-Day mortality after primary TKA	0.007	0-0.014
30-Day mortality after revision TKA	0.01	0-0.02
Wait for TKA	2 years	3 weeks to 5 years
Costs		
Indirect costs of knee OA	$10,369	0-$20,000
Direct costs of knee OA (Non-operative Treatment Bridge)	$2,500	0-$3,883
Primary TKA	$26,865	$20,000-$30,000
Revision TKA	$35,542	$30,000-$40,000

The delayed treatment remains in the end-stage OA health-state until the predetermined wait time has expired, set as 2 years in the base case. Patients receiving the non-operative bridge can experience partial symptom relief during the waiting period, while the non-bridge patients remain at the baseline end-stage knee OA utility.

Health states were assigned a health utility and cost value. Utility values are numeric values assigned to health states annually based on the commonly accepted reference values of 1 being “full health” and 0 being “death” [23]. These values are used to estimate quality-adjusted life years (QALYs), the measure of effectiveness reported in cost-utility analyses. Utility values for health states can be derived by either direct methods, such as time tradeoff and rating scales, or indirect methods, where health status instruments such as the Quality of Well-Being Scale are mapped to a numeric value between 0 and 1 based respondent answers to various attributes. Health state utility values for a wide variety of conditions have been assessed using a combination of these methods and are available in catalogued disease-specific format to provide a basis quality of life measurements in cost-effectiveness analyses [24-26]. Cost values were assigned for the primary intervention and subsequent revision procedures as the surviving cohort transitions to alternate health states based on the assigned transition probabilities. The cycle length used in this model was one month. As the theoretical patient cohort cycles through the model, costs and utilities are accumulated on a per monthly basis over the lifetime of the cohort until all members of the cohort have died. Consistent with accepted healthcare decision analysis methods, future costs and utilities were discounted at annual rate of 3% [15-17]. The Markov decision model was used to evaluate the total accumulated costs and effectiveness, measured in QALYs, of each treatment strategy to evaluate the overall cost-effectiveness of immediate primary TKA compared to delayed primary TKA for end-stage knee OA as the primary outcome in this patient cohort analysis.

### Non-arthroplasty bridge

Several non-arthroplasty bridge-to-TKA treatment options were examined. They were assumed to provide some symptomatic relief, but to fall short of that achieved by primary TKA. These consisted of corticosteroid injection, viscosupplementation, physical therapy, and non-steroidal anti-inflammatory drugs including both non-selective and COX-2 inhibitors. In the base case, patients treated with a non-operative bridge were assumed to experience a 33% improvement in utility for the entire delay before TKA. Alternative outcomes including full symptomatic relief for one quarter of the waiting period were examined with sensitivity analysis.

### Decision model assumptions

Several important assumptions were made in the construction of this model and require identification. First, we assumed the patient population defined in the model to be a theoretical cohort of healthy patients aged 60 years with end-stage knee OA treated non-operatively prior to possible TKA. This cohort is assumed to have already undergone and failed appropriate non-operative measures as outlined by the American Academy of Orthopaedic Surgeons Clinical Practice Guidelines on Treatment of Osteoarthritis of the Knee (Non-Arthroplasty) [27]. Second, we assumed the non-operative bridge would consist of treatments at the average cost for OA as measured by the Medical Expenditures Survey. Third, we assumed the non-operative bridge would not avoid the need for TKA as these patients had already failed all non-operative treatment and were considered candidates for primary TKA. Fourth, we assumed the all-cause mortality of patients in the theoretical cohort following recovery from a surgical intervention to be equal to that of the general population. Fifth, we did not directly model post-operative complications. We assumed all patients recover to the average outcome from TKA at the end of the first year and cannot undergo revision until 1 year post-op. The cost values used in the model included all costs 90 days from surgery, which would incorporate costs of complications, reoperations and readmissions.

### Model parameters

#### Population

We used a Markov decision model to analyze a theoretical cohort of 60-year-old patients with knee OA. The cohort age was set at 60 years consistent with the mean age from our literature review (Table 1) [2,6].

### Mortality rates

The probability of perioperative death for patients undergoing TKA has been used in prior publication, which was estimated from a study of the Medicare population as 90-day mortality of 0.07 with revision knee replacement at 0.11 [28]. Life expectancy and all-cause mortality rates were obtained from age-specific life tables [29].

### Utilities

Utility values for each health state were assigned based on heath-related quality of life outcome. End-stage arthritis has consistently been shown to have a utility value near 0.6 [24-26]. We defined 100% improvement from non-arthroplasty treatment as 0.9. This is consistent with other arthroplasty studies and utility studies on recovery from knee pain [22]. We assigned utility values for TKA and revision TKA that have been validated and are consistent with prior publications in the orthopaedic literature [10,12,21,22]. Utility values used for all health states in the model are shown in Table 1.

“Disutility” values represent the short-term negative impact an intervention has on a patient’s quality of life [23]. With surgical procedures, this can include pain, immobility, and non-lethal surgical complications in the post-operative and recovery periods. These transient periods of disutility are accounted for as a one-time deduction from the health-related quality of life value gained by the patient in the year of procedure. We calculated a disutility value using the time to recovery and quality of life during recovery. Our calculated values were similar to those used in previous Markov decision analyses [22,30] with TKA -0.1, revision due to infection -0.2, and revision TKA due to a disutility of -0.15. Disutility values used in the model are also shown in Table 1. No disutility for non-arthroplasty treatment was modeled.

### Costs

We used 2009 5% Medicare claims data to compute the direct medical cost of surgical treatment. We used ICD-9 diagnosis code “715.x6” to identify patients admitted to a hospital with a primary diagnosis of osteoarthritis of the knee. The following ICD-9 procedure codes were used to identify the relevant surgical techniques: “81.54” for total knee arthroplasty and “81.55,” “00.80,” “00.81,” “00.82,” “00.83,” and “00.84” for revision knee arthroplasty. The direct model costs include inpatient cost, physician cost and the costs from post-acute care facilities, such as skilled nursing facility, hospice, home health service, inpatient rehabilitation facility and long-term care hospitals. For post-acute care costs, we first estimated the percentage of patients discharged to different post-acute care facilities and the average cost of each type of post-acute care facility. We then compute the weighted average cost across all post-acute care facilities. These costs were tracked for 3 months after surgery. Additionally, the costs of 18 physical therapy visits were included as part of rehabilitation costs associated with TKA or TKA Revision. These costs, based on a Medicare population were adjusted to a private payer population.

The cost for the non-operative treatment bridge was taken from the Medical Expenditures Survey [31]. The number and type of treatments are likely to vary widely in actual practice and the mean societal value was felt to be an appropriate approximation. Lastly, in scenario B we included indirect costs associated with end-stage knee OA and after primary TKA. These include items such as lost earnings and productivity, caregiver costs, etc. These values were also taken from the MES [31]. Patients were assumed to recover 80% of the indirect costs of end-stage knee OA after successful primary TKA [32]. These costs can be found in Table 1.

### Cost-effectiveness analysis

The Markov model was used to conduct a cost-effectiveness analysis of the decision process for non-arthroplasty treatment of knee OA. The present-day value of the expected costs and QALYs gained over the lifetime of a theoretical patient cohort was calculated based on treatment strategy. Outcome measures included average costs and effectiveness (QALYs), as well as the cost-effectiveness (C/E) ratio for each strategy. The incremental costs and effectiveness were also calculated and represent the relative difference between the two alternative strategies. The principle outcome measurement calculated was the incremental cost-effectiveness ratio (ICER), which is the ratio between the difference in costs and difference in QALY of each strategy. In terms of this model, the ICER could be expressed as ICER = (Cost_TKA without delay_ – Cost_delayed TKA_) / (QALYs_TKA without delay_ – QALY_delayed TKA_). ICERs less than $50,000 per QALY gained were considered to be cost-effective based on a willingness of the health-care system to pay (WTP) value of $50,000. In this cost-effectiveness analysis, the preferred treatment strategy was the most effective strategy with an ICER < WTP.

One, two and three way sensitivity analyses were performed on all variables in the model. Variables deemed sensitive are those who when variable across a reasonable range change the preferred strategy. If the preferred strategy does not change, then the variable is termed robust.

This study was approved by the Duke University Health System’s Institutional Review Board.

## Results

### Base case

In the base case, a 2-year wait-time both with and without a non-operative treatment bridge resulted in a lower number of average quality-adjusted life-years gained (11.57 QALY (no bridge) and 11.76 QALY (bridge) vs. 12.18 QALY (no delay). The average cost to society was $2,920 higher for TKA without delay than a 2 year wait-time with no bridge, but $2,143 less than a wait-time with a non-operative treatment bridge. The incremental cost effectiveness ratio comparing wait-time with no bridge to TKA without delay was $4,768/QALY, below the willingness-to-pay threshold of $50,000/QALY. When comparing TKA without delay to waiting with a non-operative bridge, TKA without delay produced greater utility for the patient at a lower cost to society, therefore, dominating the waiting with non-operative bridge strategy for the treatment of knee osteoarthritis.

However, when indirect costs are included, TKA without delay produces greater utility at a lower cost to society than either wait-time strategy. The incremental cost of delayed TKA with a non-operative bridge rises to $18,900 and $13,836 for delay without the treatment bridge. Utility remains the same. The results of the base case are found in Table 2.

**Table 2:** Results of analysis for base case

**Cost scenario**		**Avg. cost**	**Avg. QALYs gained**	**Avg. cost difference**	**Avg. difference in QALYs gained**	**Cost-effectiveness ratio**	**Incremental cost-effectiveness ratio**
A	TKA without delay	$17,840	12.18	$1,667	0.61	$1,464/QALY	$2,723/QALY
	Delayed TKA with Nonop Bridge	$21,230	11.76	$3,398	0.19	$1,806/QALY	$17,880/QALY*
	Delay + No Bridge	$16,170	11.57	-	-	$1,398/QALY	-
B	TKA without delay	$59,640	12.18	-	0.61	$4,897/QALY	
	Delayed TKA with Nonop Bridge	$78,541	11.76	$18,900	0.19	$6,679/QALY	DOMINATED**
	Delay + No Bridge	$73,477	11.57	$13,836	-	$6,351/QALY	DOMINATED**

Cost scenario A = Direct costs only, Cost scenario B = Indirect costs included

*Waiting with a non-operative bridge resulted in a lower number of average quality-adjusted life-years gained while also at a higher average cost to the payer and is, therefore, “DOMINATED” by the TKA without delay strategy for the treatment of end-stage knee osteoarthritis in the base case. It is considered cost effective compared to delayed TKA with no treatment bridge.

**When indirect costs are considered, TKA without delay is both less costly and more effective than the other two strategies and is therefore a dominant treatment strategy.

### Sensitivity analysis

Multivariate sensitivity analyses demonstrated that all variables were robust with cost effectiveness as the outcome measure. Only when the utility of primary TKA falls below the utility of knee pain would either of the delayed scenarios become preferred. When considering cost as the outcome, if a patient experiences $151.57/month or greater (base case = $857) of indirect costs such as lost wages or caregiver expenses, TKA without delay becomes less costly and, therefore, a dominant treatment strategy. Similarly, if the cost of the non-operative bridge falls below $294/month, it becomes less costly than TKA without delay, but TKA without delay is still the preferred cost effective strategy. When evaluating the non-operative bridge only compared to no treatment, if the patient receives 13 months or more of symptomatic relief with the bridge while waiting for a TKA, then the bridge is the preferred cost effective strategy if TKA without delay is not available. The ability of TKA to reduce the indirect costs associated with end-stage knee OA is an important variable – TKA without delay is less costly than a 2 year wait-time when TKA reduces 15% or more of these indirect costs.

**Figure 2:**
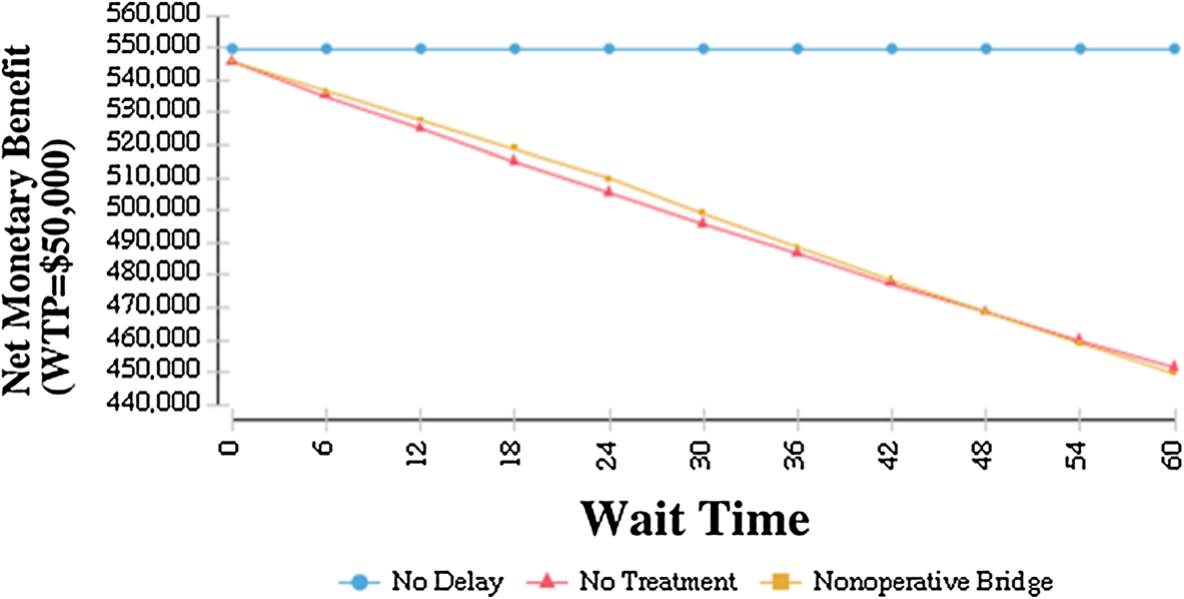
**Sensitivity analysis on potential wait times with net benefits as outcome.** The graph shows the results of a one-way sensitivity analysis on wait times up to 60 months for primary TKA with net benefits as the outcome.

**Figure 3:**
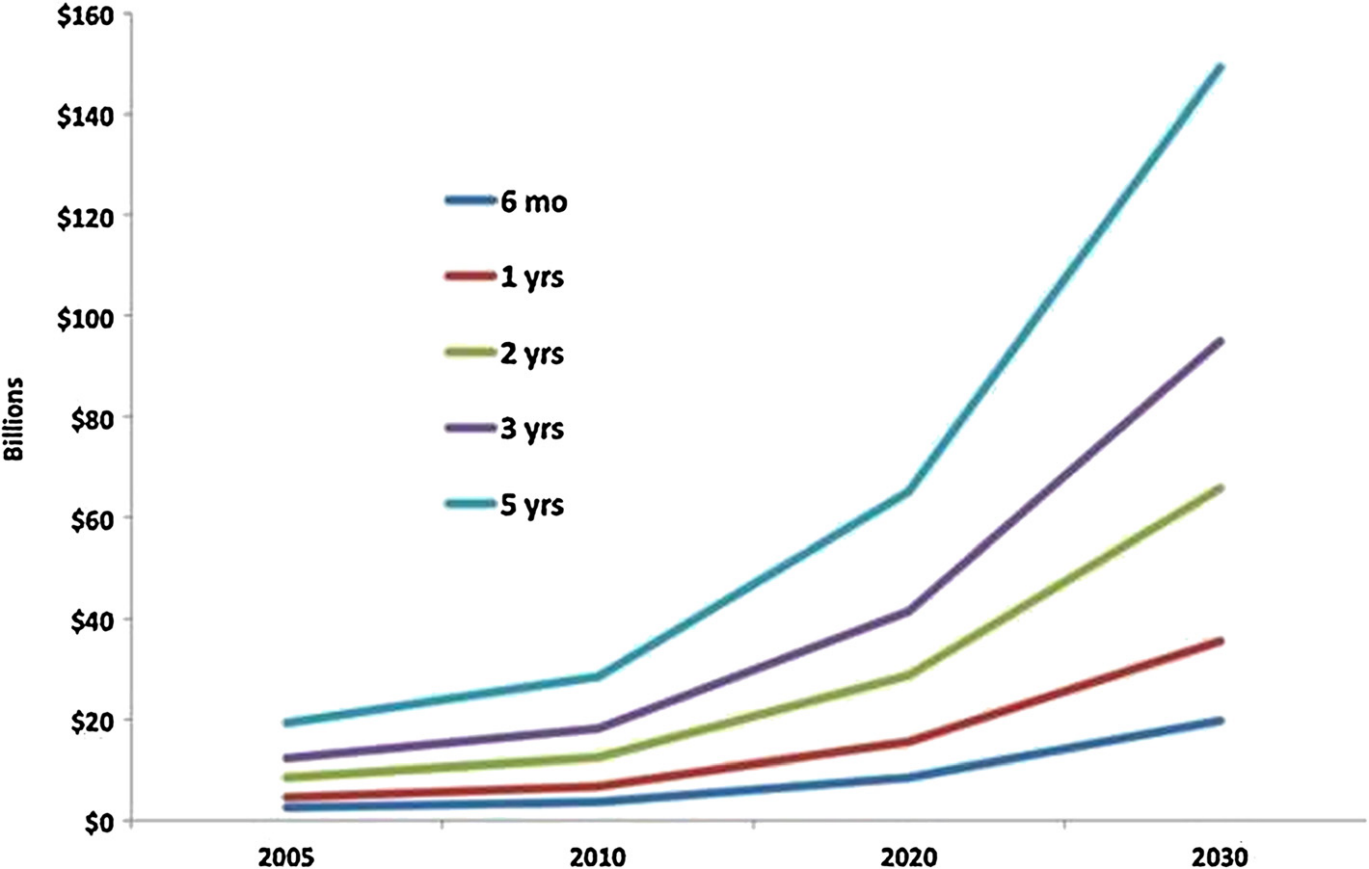
**Societal economic impact of potential wait times.** The figure shows the estimated future societal costs of delayed TKA. Values are costs only and do not include monetary value of utility. These values represent the incremental cost of each wait time with non-operative treatment bridge compared to TKA without delay baseline. They are extrapolated from data from out model combined with volume projections from Kurtz et al. [3].

Figure 2 shows the effect of increasing wait times on net health benefits. As wait times increase, net health benefits fall precipitously. In Figure 3, the future societal costs of delayed TKA are estimated using the projections established by Kurtz et al [3]. The cost scenario using indirect costs for the strategy delayed TKA with non-operative bridge is shown. Per patient, for a wait time of 6 months and 5 years, the incremental cost is $5,652 and $42,832 respectively. In the year 2020, this translates to an incremental cost of $8.59 billion for a wait time of 6 months. If the wait time were to soar to 5 years, the incremental cost in 2030 would reach nearly $150 billion (149.1). The impact on quality of life is substantial as well – in the year 2020 for a wait time of 2 years, TKA without delay results in an additional 927,200 QALYs or years of perfect health related to end-stage knee OA.

## Discussion

Delaying primary TKA for patients who have failed all non-operative treatments for end-stage knee OA appears to be an inefficient strategy. Immediate primary TKA for end-stage knee OA is the preferred cost effective strategy concerning the timing of the delivery of primary TKA. Compared to the 2-year waiting period modeled in the base case, TKA without delay was a dominant treatment strategy when indirect costs are included or when a non-operative bridge costing over $87 per month is used during the waiting period.

Extrapolating these costs to the estimates for the demand for primary TKA shows staggering results. Within 10 years expanding wait times could be responsible for an additional $10-$80 billion. Within 20 years this number could reach $84 billion in annual incremental costs using the base case wait time of 2 years. In the worst case scenario of a 5-year wait time, incremental costs could approach $200 billion.

Wait times for primary total joint arthroplasty have already begun to rise. The wait time in Canada has grown by 437% since the mid 1990s and is now over 6 months, the critical period where health-related quality of life is adversely affected. The problem extends beyond orthopaedic surgery and the U.S. – In a 5 country analysis in 2002, Blendon et al. reported that the percentage of patients waiting greater than 4 months for any elective surgery increased by at least 4% during a 3 year period in all 5 major countries that he examined [33]. Wait times in the U.S. have not been examined recently but were measured at only 3 weeks in 1994.

The impact of these findings suggests that policymakers should make concerted efforts to support the development of innovative strategies and expansion of existing ones to meet demand. Increasing the size of the workforce has been discussed and projections in Canada suggest that the annual supply of surgeries must increase by 12% or greater each year to prevent further increases in mean waiting times [34]. Payment reform must also be carefully crafted to incentivize appropriate utilization. Lastly, decisions must be made with consideration of the entire healthcare system and avoid too narrow of a focus on one disease.

The weaknesses of this study include primarily the estimation of costs. We utilized Medicare reimbursement, an accepted approach for cost effectiveness analysis. This approach may not capture the complete cost of care, but sensitivity analysis revealed the cost of primary TKA was robust. As such, the inaccuracies of cost estimation would not affect the preferred treatment. Greater uncertainty surrounds the reduction in indirect costs after TKA. While the indirect costs of end-stage OA are well-studied, the ability of TKA to reduce these costs is less known. Several studies have investigated return to work after primary total joint arthroplasty [35], but only one examined TKA and reported a return to work after 30-day rate of 82% [29]. In the base case we assumed an 80% reduction in indirect costs, but rigorous sensitivity analysis was performed, demonstrating that TKA without delay becomes less costly when 15% or more of these costs are affected. As this number is well below the lowest number reported in the literature, the reduction in indirect costs after primary TKA is practically robust. However, this assumption needs further study to provide a more accurate projection of the total societal cost of wait times.

## Conclusions

In summary, minimizing wait times for primary TKA is cost effective and may be cost saving. It clearly increases utility for the patient, but the cost implications are less clear due to estimates of indirect costs. However, the threshold of indirect cost where TKA is cost saving assuming a 2-year wait time is low and well below most published estimates. Policymakers cannot ignore the societal impact of diminished access to total knee arthroplasty.

## Abbreviations

TKA: Total knee arthroplasty; OA: Osteoarthritis; QALY: Quality-adjusted life year; ICER: Incremental cost-effectiveness ratio.

## Competing interests

The authors declare that they have no financial competing interests. The authors declare that they received no external funding for support of this study. One of the authors (RCM), served as a Washington Health Policy Fellow from 2010-2011 for the American Academy of Orthopaedic Surgeons.

## Authors’ contributions

RCM conceived of the study, participated in its design and coordination, conducted the Markov modeling analysis, helped conduct the statistical analysis, and helped to draft the manuscript. KTH performed the background research and literature review for the study, and helped to create the figures and tables. LAO participated in the design of the study, conducted the statistical analysis, and helped to draft the manuscript. TSW conceived of the study, created the Markov model for analysis, performed the background research and literature review, and helped to draft the manuscript. LK performed the background research for the study and helped conduct the statistical analysis. RMN conceived of the study, participated in its design and coordination, and helped to draft the manuscript. MPB conceived of the study, participated in its design and coordination, helped to create the Markov model, and helped to draft the manuscript. All authors read and approved the final manuscript.

## Pre-publication history

The pre-publication history for this paper can be accessed here:

http://www.biomedcentral.com/1471-2474/15/22/prepub
